# Understanding the Pathogenesis, Biocontrol Mechanisms, and Factors Influencing Biocontrol Effectiveness for Soil-Borne Diseases in *Panax* Plants

**DOI:** 10.3390/microorganisms12112278

**Published:** 2024-11-10

**Authors:** Zhaobei Wang, Shuoye Wang, Hongyan Yang

**Affiliations:** College of Life Sciences, Northeast Forestry University, Harbin 150040, China; 198337610267@163.com (Z.W.); 18731933800@139.com (S.W.)

**Keywords:** *Panax*, soil-borne disease, pathogen, biocontrol, microbial diversity, biocontrol effectiveness

## Abstract

*Panax* plants are known for their significant medicinal and economic value. Being perennial, they are prone to soil-borne diseases during cultivation. However, there has been limited research on the pathogenesis of soil-borne diseases and the diversity of pathogens. While biological control has gained attention for its efficacy and environmental benefits, the factors affecting its efficiency still need thorough evaluation. This review summarizes the influence of biotic factors, such as pathogens and hosts, and environmental factors on the occurrence of soil-borne diseases and pathogen diversity. Additionally, we synthesized bacterial, actinobacterial, and fungal diversity for the biocontrol of soil-borne diseases and their functional mechanisms. Moreover, the review delves into the factors influencing the efficacy of biocontrol, including microbial species, the inoculation method and inoculation volume, and inoculant composition. This article serves as a valuable resource for enhancing the efficiency of biological control and optimizing strategies for managing soil-borne diseases in *Panax* cultivation in the future.

## 1. Introduction

The *Araliaceae* family encompasses a wide array of plants, consisting of over 1500 species [1], with 114 of these species having medicinal properties that hold significant importance for human health. Among these *Araliaceae* plants, *Panax* species, such as *Panax ginseng*, *P. quinquefolium*, *P. notoginseng*, *P. japonicum*, and *P. trifolium*, possess valuable medicinal attributes, including tumor inhibition, blood lipid reduction, antithrombotic effects, promotion of blood circulation, and removal of blood stasis [2,3]. Moreover, *Panax* plants have been widely used as raw materials for healthcare products, cosmetics and dietary supplements [4]. For example, *Panax ginseng* alone is projected to reach a global trade volume of around $17.7 billion by 2030 [5]. Consequently, the market demand for these plants continues to grow, necessitating large-scale cultivation. However, their perennial nature renders them susceptible to frequent occurrence of soil-borne diseases, which significantly impacts the yield and quality of the plants [6].

Soil-borne diseases are plant diseases that spread through soil [7]. Soil-borne pathogens could be bacteria, fungi, or viruses and can cause different plant diseases. It is widely recognized that fungi are the primary pathogens responsible for soil-borne diseases in *Panax* [8,9], causing root or foliar diseases [10,11]. Understanding the diversity of pathogens is crucial for effective prevention and control of soil borne diseases [12]. Regrettably, there is currently a lack of systematic knowledge regarding pathogen diversity of *Panax* plants. Additionally, when a disease occurs, in addition to pathogens, there is a lack of knowledge regarding the susceptible host and favorable environments that allow the pathogens to thrive [7]. However, a comprehensive summary of these factors specific to *Panax* plants’ soil-borne diseases is currently lacking.

Traditionally, chemical control has been widely used in the management of soil-borne diseases. However, the extensive use of pesticides not only causes cost increases, drug residues, environmental pollution, and pathogen resistance, but also affects soil microecological balance [13]. Biological control, on the other hand, is an important alternative to chemical control, as it provides an effective and environmentally friendly strategy for managing soil-borne diseases [14]. Studies have reported that biocontrol microorganisms play a crucial role by either antagonizing pathogens or regulating plant disease resistance [15]. Understanding and analyzing the biocontrol mechanisms against pathogens is of great significance in developing more efficient biocontrol strategies. However, the exploration of soil-borne disease biocontrol in *Panax* plants is still limited and requires further investigation to enhance our understanding.

In this study, we firstly present a comprehensive summary of soil-borne disease pathogenesis and the diversity of pathogens in *Panax* plants. Then, we hypothesize that biological control is effective in managing soil-borne diseases and that biological control factors play a significant role in disease control. We focus on antagonistic microbial diversity, biocontrol mechanisms employed by antagonistic microorganisms, and systematically assess various factors in relation to soil-borne diseases’ biocontrol efficiency, aiming to provide a technical reference that can enhance the effectiveness of biocontrol measures and optimize strategies for managing soil-borne diseases in the future.

## 2. Pathogen Diversity of Soil-Borne Disease in *Panax* Plants

All data are based on published data regarding *Panax* plants from 1995 to 2024 from Web of Science, PubMed, and Google Scholar. To clarify the pathogen diversity, we used three sets of keywords, “*Panax*” and “root-rot” (or “blight” or “black-spot”), to search the relevant literature on soil-borne diseases. A total of 268 references about pathogen diversity were obtained.

**Figure 1:**
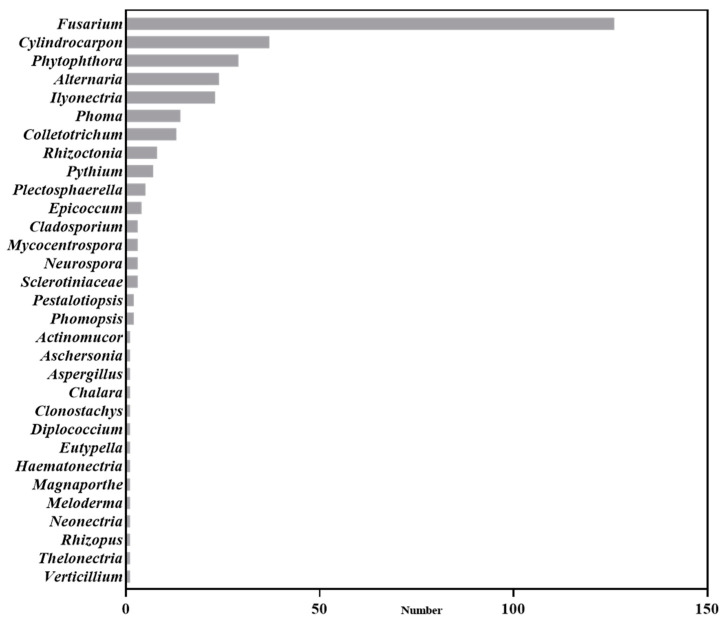
Soil-borne disease pathogens reported in *Panax* plants.

Based on an analysis of current publications in the field of *Panax* plants, our findings reveal that *Panax* plant pathogens encompass thirty-one fungal genera, mainly including *Fusarium*, *Cylindrocarpon*, *Ilyonectria*, *Alternaria,* and *Phytophthora* (Figure 1). A substantial portion of the research has focused on *Fusarium* (37.5%), *Cylindrocarpon* (11.01%), and *Phytophthora* (8.63%). The above pathogens did not only exist for *Panax* plants. For example, *Fusarium* can infect various plants, causing root rot, wilt, or necrosis [16]. But they all infect *Panax* plants [17,18], and some *Panax*-specific pathogens were also found. For example, the *P*. ginseng-specific type of *F. oxysporum* was studied in 2022 [19]. *Cylindrocarpon* and *Ilyonectria* are known as both root rot and rust rot pathogens [20]. Among the *Cylindrocarpon* species, *C. destructans* has been reported especially in regard to *Panax* plants [21]. Although there are no *Panax*-specific *Phytophthora*, *Phytophthora cactorum* has caused both root and foliar diseases [22] in *Panax* plants.

## 3. Pathogenesis of Soil-Borne Diseases in *Panax* Plants

After wintering in resting soil, soil-borne pathogens could spread to the plants in the next season. Disease occurrence involves the following aspects.

### 3.1. Autotoxin Secretion of Panax Plants

Autotoxicity in plant species is a phenomenon where intraspecific allelopathy occurs. It involves the release of compounds such as phenolic compounds, terpenoids, and nitrogenous organic compounds [23]. *Panax* plants, however, exhibit a distinct characteristic of continuously accumulating saponins, which are secondary metabolites known for their key active components extensively used in food, healthcare products, and pharmaceuticals [24]. Saponins play a crucial role in inducing disease occurrence, potentially aiding in the growth of pathogens [25]. Pathogens reported, such as *Pythium cactorum*, *P. irregulare*, and *Cylindrocarpon destructans,* display a strong chemotaxis towards saponins [26]. They are capable of rapidly degrading or utilizing saponins as growth-promoting factors to facilitate their invasion and reproduction. It is reported that ginsenosides belonging to propanaxanol group can promote *C. destructanans* growth by nearly 130% [27].

### 3.2. Toxic Effects of Pathogen

When the condition is appropriate, the hyphae or conidia of pathogens in soil begin to infect from the roots. Some of them directly attack the roots, causing disease from the roots, which could lead to *Panax* plants’ withering and death, such as *Fusarium* and *Cylindrocarpon* [20]. Alternatively, some pathogens can colonize into the root and invade the whole plant, leading to disease occurrence in the aboveground sections of *Panax* plants, such as wilting or spot disease, including pathogens Alternaria and Phytophthora [28,29]. While invading, pathogens could secrete virulence factor (VF), which is a pathogen-produced factor that causes diseases [30,31]. In the case of *Panax* plants, studies on *F. oxysporum* and *Ilyonectria morpanacis* have reported the functions of VF. In *F. oxysporum* infection, the pathogen first secretes fusaric acid, which accelerates colonization. It then invades the plant’s exosomes by producing cell wall degradation enzymes, thereby compromising the plant’s immune system [32,33]. For *I. morpanacis* infection, it also rapidly secretes substantial quantities of hydrolases such as cellulase and pectinase, which aid in the rapid invasion of the epidermis and the subsequent spread through the cortex and internal tissues [20].

### 3.3. Environmental Factors

Apart from plant and pathogen factors, abiotic factors also play a significant role in root rot. Environmental changes can create a favorable environment for pathogen growth and increase the susceptibility of plants to pathogenic infections [20]. Temperature and humidity particularly play a crucial role in root rot occurrence, as the disease tends to thrive in seasons characterized by high temperatures and humidity [34]. A study conducted on *P. notoginseng* has confirmed temperature and humidity as the main factors influencing root rot [8]. Additionally, soil pH has a profound impact on the severity of root rot. The disease is more likely to occur in acidic soils with a pH below 5. This is supported by direct evidence from findings involving *C. destructans*. The development of root lesions was significantly reduced at pH 7.0 in comparison to a pH of 5.0 [35].

## 4. Microbial Diversity for Biocontrol of Soil-Borne Diseases in *Panax* Plants

Biological control is an environmentally friendly approach to plant disease management that utilizes live microorganisms to regulate soil microecosystems, effectively safeguarding plants against pathogenic microorganisms and guiding the progression of the microbial community towards balanced succession [10]. Bacteria, actinomyces, and fungi have all been used for biological control of plant soil-borne diseases.

In terms of bacteria, *Bacillus* spp., *Pseudomonas* spp., and *Burkholderia* spp. are the most commonly employed for disease control in *Panax* plants, given their potent antagonistic effects. For instance, *Bacillus amylophilus* AK-0 has been found to significantly inhibit the growth of *C. destructans* [36]. Similarly, *B. velezensis* has demonstrated inhibitory effects against *F. oxysporum* [23]. *P. aeruginosa* strain D4 effectively combats *Ilyonectria* sp., *Cladosporium* sp., *Aschersonia* sp., and *Fusarium* sp. [37]. Moreover, *Burkholderia* sp. has shown remarkable inhibition of *Fusarium* and *C. destructans* [38,39]. In addition, *Brevundimonas* and *Paenibacillus* have exhibited strong antagonistic effects on *Alternaria* spp. and *F. oxysporum* [11,40,41].

Actinobacteria, as the primary sources of antibiotics [42], have been discovered to possess the ability to control soil-borne diseases, including gray mold [43] and rust rot [44] in *Panax* plants. In terms of root rot control, *Streptomyces* has displayed exceptional efficacy [45]. For instance, bioactive substances extracted from *S. cellulosae* YIM PH20352 exhibited effectiveness against the pathogen *Alternaria* in *P. ginseng* [46]. Additionally, Huang et al. identified that *Streptomyces* G7 produced polyketide lydicamycinsand other active metabolites, inhibiting pathogenic organisms such as *F. graminearum*, *Ustilaginoidea virens* and *Magnaporthe oryzae* pathogenic to plants [47].

Among the fungi, *Trichoderma*, *Chaetomium,* and *Penicillium* have been found to effectively control soil-borne diseases in *Panax* plants [48,49]. *Trichoderma* has been employed to manage those caused by *Phytophthora. cactorum* in *P. notoginseng* [50]. *Chaetomium*, on the other hand, demonstrates inhibitory effects against root rot pathogens such as *F. flocciferum*, *Phoma herbarum* and *Plectosphaerella cucumerina*, which are isolated from *P. notoginseng* [51]. Moreover, *Penicillium* has proven effective in controlling root rot caused by *F. oxysporum* in *P. ginseng* [52]. Recently, *Mortierella* has been identified as having the ability to enhance the resistance of *P. ginseng* against root rot [14].

## 5. Biocontrol Mechanisms of Soil-Borne Disease Suppression

**Figure 2:**
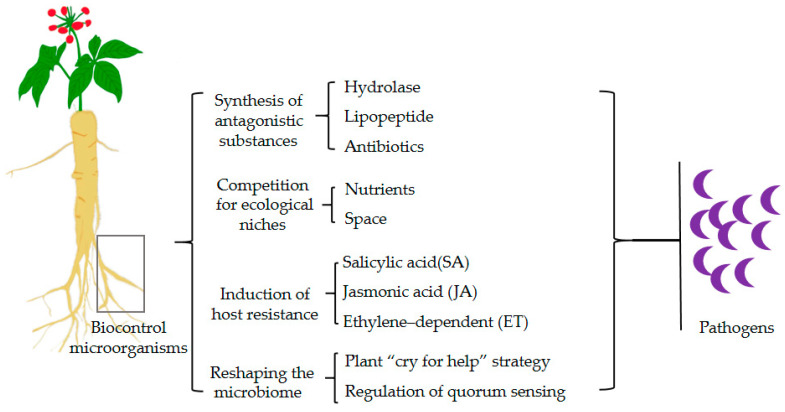
Biocontrol mechanisms of soil-borne disease suppression.

In the context of soil-borne diseases, biocontrol microorganisms can operate through various mechanisms, including the synthesis of antagonistic substances, competition for ecological niches, and the induction of host resistance (Figure 2). The typical microorganisms and their mechanisms are listed in Table 1.

### 5.1. Synthesis of Antagonistic Substances

Biocontrol microorganisms possess the ability to directly inhibit pathogens through the secretion of antagonistic substances, including both non-volatile and volatile compounds. Non-volatile substances, such as hydrolases, lipopeptides, and antibiotics play a significant role in this inhibition. Hydrolases could degrade the cell walls of a number of phytopathogenic fungi [63]. For instance, *Paenibacillus polymyxa* SY42 produces cellulase and protease to effectively degrade the cell wall of *F. oxysporum*, thus protecting plants against infection [53]. Similarly, *Actinomyces* BX5, BX17, and BX26 hinder the growth of *F. graminis* by targeting the cell wall integrity using hydrolases [60]. Lipopeptides can function by the cell membrane and form aggregates with phospholipids, thereby changing the permeability of the cell membrane of target pathogens [64]. Taking *B. amyloliquefaciens* FS6 as an example, it can inhibit *F. solani* in *P. ginseng* [65]. Antibiotics function by comprehensive mechanisms. Recently, a new antibiotic echinosporin from *Amycolatopsis* showed antifungal activity against root-rot pathogens such as *F. oxysporum* and *Alternaria panax* of the *P. notoginseng* [66].

Volatile organic compounds (VOCs) possess unique characteristics such as low molecular weight, low boiling points, and significant volatility, enabling them to disperse into the atmosphere and soil, thereby directly inhibiting pathogens [67,68]. The main mechanisms underlying the antifungal effects of VOCs are the disruption of cell walls and membrane structures, leading to intracellular lysate leakage and oxidative stress induction [69]. Research has shown that VOCs produced by *B. subtilis*, *S. setonii,* and *Nocardiopsis* sp. exhibit inhibitory effects on *Curvularia lunata* in maize, *Ceratocystis fimbriata* in sweet potato, and *Ganoderma* sp. in palm, respectively [70,71,72]. In *Panax* plants, two studies have reported the inhibitory effects of VOCs, with the VOC of *T. koningiopsis* T-403 inhibiting *C. destructans* by 84% [59] and the VOC from *B. velezensis* W17 inhibiting *F. oxysporum* by 31.89% [23].

### 5.2. Competition for Ecological Niches

Niche competition refers to the competitive interactions that take place between individuals as they vie for limited resources, such as food, space, and light. Competition for nutrients and space is a prominent manifestation of niche competition [73]. Biocontrol microorganisms have the capacity to suppress pathogens by engaging in competition for these crucial nutrients and space. For instance, *Streptomyces* could compete with *Fusarium* for resources and nutrients [61]. *Chaetomium globosum* LB-2 significantly inhibits the growth of *F. oxysporum* and *Exserohilum turcicum,* potentially through competition for resources [74]. The successful colonization of biocontrol microorganisms on the root surface is a crucial step in repelling soil-borne pathogens from occupying ecological spaces and invasion sites [75]. For example, *Bacillus* can colonize tissues, occupy space, and deplete nutrients before pathogen infection, thus gaining competitive advantages [76,77,78]. Biofilm formation on roots by *Bacillus* agents contributes to spatial competition with plant pathogens and ultimately suppresses disease development [79,80].

Iron is an essential micronutrient that is present in a high percentage of soils; however, it has low solubility in soil with a pH > 6 and is not suitable for uptake by microorganisms [81]. Therefore, iron bioavailability usually becomes a limiting factor that causes nutrient competition among living microbes [82]. Some strains of *Bacillus* spp. and *Pseudomonas* spp. can compete with pathogens for iron by producing siderophores, which effectively chelate the available iron (Fe) in the plant, thus depriving the pathogen of this vital nutrient [54]. Due to iron deficiency, pathogenic fungal spore germination is inhibited and hyphal growth restrained, effectively lowering the chance that the plants become infected, and reducing disease incidence and severity [83].

### 5.3. Induction of Host Resistance

Systemic resistance is triggered by necrotizing pathogenic microorganisms as well as non-pathogenic rhizobacteria, providing protection against a wide range of pathogens [84]. There are two types of systemic immunity studied in the context of local plant-microbe interactions: systemic acquired resistance (SAR) and induced systemic resistance (ISR), which depend on the site of induction and the lifestyle of the inducing microorganism. SAR is induced by pathogens that interact with plant leaves, while ISR is elicited by beneficial microorganisms that interact with plant roots [85]. For instance, the interaction of some *Bacillus* strains with plant roots elicits ISR and enhances the resistance of the entire plant against pathogens [55]. ISR mediated by biocontrol microorganisms can trigger the up-regulation of defense genes in *P. ginseng*, such as *PgPR5*, *PgPR10*, *PgCAT*, ultimately inducing systemic resistance [86,87]. Among actinomyces, *Streptomyces* AcH 505 is another example that can induce plant resistance to *Erysiphe necator* by activating salicylic acid (SA) and jasmonic acid (JA)/ethylene–dependent (ET) signaling pathways [62]. Among fungi, *Trichodema citrinoviride* can enhance *P. ginseng* resistance against *B. cinerea* by up-regulating the expression of defense-related genes, including *PR2, PR4, PR5,* and *PR10* [58].

### 5.4. Reshaping the Soil Microbiome

Reshaping the structure and function of the soil microbiome can lead to the suppression of plant diseases, which could be performed by the plant “cry for help” strategy and the regulation of quorum sensing (QS).

When pathogens attack, plants will develop “cry for help” strategies to attract beneficial microorganisms to the rhizosphere or roots, thus aiding in disease resistance [88]. The “cry for help” response represents a potential mechanism through which rhizosphere microbiota actively inhibit disease progression [89]. It has been reported that *P. notoginseng* recruits potentially beneficial microorganisms with disease-inhibiting functions, such as *Sphingobium*, *Pseudoxanthomonas*, *Pseudomonas*, *Stenotrophomonas*, and *Flavobacterium* into the rhizosphere. Increasing relevant biocontrol microorganisms could serve to combat the invasion of *F. oxysporum*, *F. solani*, and *Ilyonectria* pathogens [57]. In *P. notoginseng,* it has been documented that probiotic consortia consisting of eight microbial species can alter the soil microbiota and inhibit root rot disease [56].

QS might be an effective mechanism by which various microorganisms can regulate their gene expression and accordingly synchronize their biological behavior according to their population density. Among Gram-negative bacteria such as *Pseudomonas*, the most widely reported QS signal is acylhomoserinolactone (AHL) [90]. It has been proved that, for *P. ginseng*, the use of AHL can reshape the soil microflora and offers potential for promoting growth and enhancing resistance [91].

Understanding biocontrol mechanisms, combined with the pathogenesis of soil-borne diseases, will aid to provide guidance for better applying biocontrol microorganisms, improving biocontrol effectiveness and formulating better biocontrol strategies [32].

## 6. Factors Influencing Biocontrol Efficacy in *Panax* Plants

Biological control has been proved to be a successful approach in the prevention of soil-borne diseases. However, there is currently a lack of systematic investigations on the factors influencing the effectiveness of biocontrol in *Panax* plants. To address this knowledge gap, based on publications as described as Section 2, we further scrutinized related studies. Inclusion was based on four screening criteria: (1) the study’s focus on the efficacy of biological control against soil-borne diseases in *Panax* plants (excluding reviews and meta-analyses), (2) the presence of experimental and control groups, (3) the use of disease incidence (DI) or the disease severity index (DSI) to assess the effects of biological control, and (4) considering results from different trials within the same article as independent observations. Data from tables and article descriptions were directly extracted, whereas data from figures was extracted using GetData Graph Digitizer 2.20 (https://getdata-graph-digitizer.software.informer.com/2.2/, accessed on 6 November 2024). Subsequently, the decrease rate was calculated based on the means of DI or DSI, according to the formula: control efficacy (%) = (DI or DSI in treatment with pathogen—DI or DSI in treatment with biocontrol microorganisms)/(DI or DSI in treatment with pathogen) × 100 [92].

### 6.1. Microbial Species Effects on Biocontrol Effectiveness

**Figure 3:**
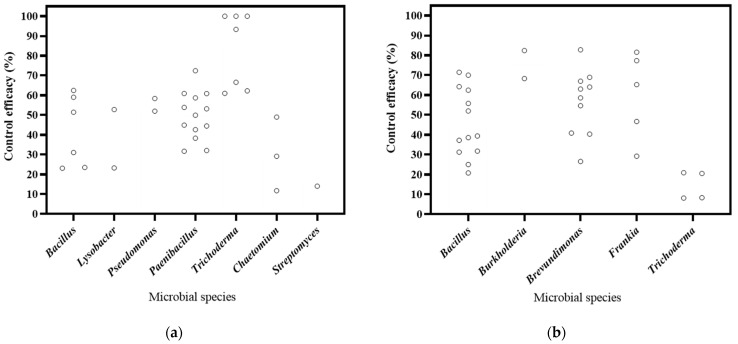
Effects of microbial species on biocontrol effectiveness. (**a**) control efficacy in disease incidence (DI) [57,58,93,94,95,96,97,98,99,100,101,102]; (**b**) control efficacy in disease severity index (DSI) [11,13,103,104,105,106,107,108,109,110].

The literature included in this study focuses on biocontrol bacteria, fungi, and actinomyces, in 15, 7, and 3 publications, respectively (Figure 3). Based on the DI, the decrease rates of bacteria, fungi, and actinobacteria ranged from 23.29% to 72.46%, from 61.01% to 100.00%, and 14.09%, respectively. The most substantial decrease was observed in *Trichoderma*. As for DSI, the decrease rates of for bacteria, fungi, and actinobacteria ranged from 25.05% to 82.77%, from 43.29% to 60.32%, and from 29.25% to 81.59%, respectively. And *Burkholderia* exhibited the highest efficacy in disease control. Therefore, the findings of this study suggest that these microorganisms have significant potential for controlling soil-borne diseases in *Panax* plants.

### 6.2. Effects of Inoculation Method on Biocontrol Effectiveness

**Figure 4:**
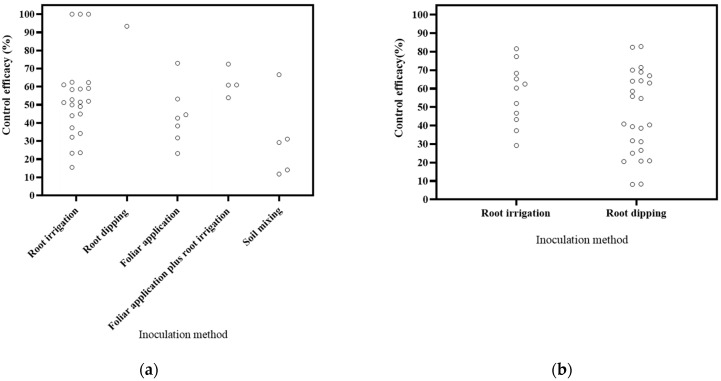
Effects of inoculation method on biocontrol effectiveness. (**a**) control efficacy in disease incidence (DI) [56,57,58,93,94,95,96,97,98,99,100,101,102,112]; (**b**) control efficacy in disease severity index (DSI) [11,13,50,103,104,105,106,107,108,109,110].

Various inoculation methods were employed, including root irrigation, foliar application, root dipping, soil mixing, and a combination of foliar application and root irrigation (Figure 4). The literature covered 16 publications on root irrigation application, 2 on foliar application, 6 on root dipping application, and 1 on foliar application plus root irrigation application. Based on the DI, root irrigation application exhibited a reduction range of 15.45–100%, foliar application showed a range of 23.15–72.95%, root dipping application resulted in a reduction of 93.34%, foliar application plus root irrigation application yielded a range of 53.89–72.46%, and soil mixing resulted in a reduction a range of 11.81–66.57%. Notably, root irrigation application demonstrated the highest effectiveness in disease control. Based on the DSI, root irrigation application led to a reduction range of 29.25–81.59%, while root dipping application showed a range of 8.19–82.77%. Root dipping application exhibited the most effective control. These results align with the commonly held assumption in soil-borne disease control that methods directly targeting the roots, such as root dipping and root irrigation, tend to be more efficient [111].

### 6.3. Effects of Inoculation Volume on Biocontrol Effectiveness

**Figure 5:**
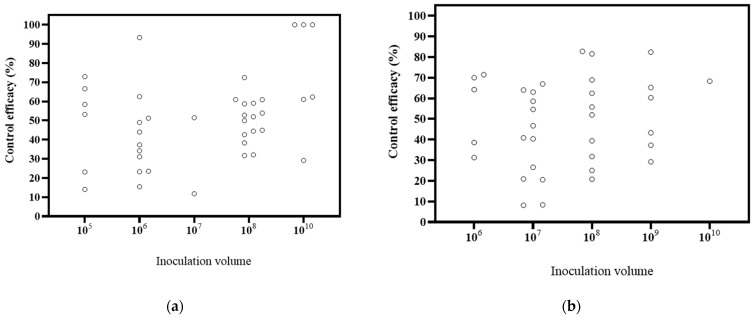
Effects of microbial volume on biocontrol effectiveness. (**a**) control efficacy in disease incidence (DI) [56,57,58,93,94,95,96,97,98,99,100,101,102,112]; (**b**) control efficacy in disease severity index (DSI) [11,13,50,103,104,105,106,107,108,109,110].

The papers included in the study covered different inoculation volumes, including 10^5^ CFU/mL, 10^6^ CFU/mL, 10^7^ CFU/mL, 10^8^ CFU/mL, 10^9^ CFU/mL, and 10^10^ CFU/mL, with respective counts of 3, 8, 5, 9, 4, and 3 publications (Figure 5). Based on the DI, the decrease ranges were 14.09–66.57% at 10^5^ CFU/mL, 15.45–93.34% at 10^6^ CFU/mL, 11.80–51.52% at 10^7^ CFU/mL, 31.74–72.46% at 10^8^ CFU/mL, and 29.21–100% at 10^10^ CFU/mL. The highest biological effectiveness was observed at 10^10^ CFU/mL. Based on the DSI, the decrease ranges of were 31.30–71.52% at 10^6^ CFU/mL, 8.19–67.00% at 10^7^ CFU/mL, 20.80–82.77% at 10^8^ CFU/mL, 29.25–82.42% at 10^9^ CFU/mL, and 68.29% at 10^10^ CFU/mL. The greatest biological control effectiveness was achieved at 10^8^ CFU/mL. These findings, combined with the DI and DSI, suggest that higher inoculation volumes tend to result in better effects. It is widely accepted that higher inoculation volumes lead to improved control effects [113]. For instance, a study suggested that inoculation volumes of 10^6^ and 10^7^ CFU/mL did not achieve the desired inhibitory activity and recommended a higher concentration for an appropriate effect [78]. The results of this study support this conclusion.

### 6.4. Effects of Microbial Composition of Biocontrol Effectiveness

**Figure 6:**
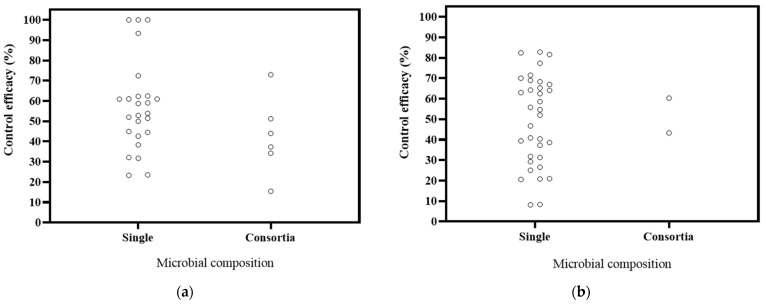
Effects of microbial composition on biocontrol effectiveness. (**a**) control efficacy in disease incidence (DI) [56,57,58,93,94,95,96,97,98,99,100,101,102,112]; (**b**) control efficacy in disease severity index (DSI) [11,13,50,103,104,105,106,107,108,109,110].

The literature on microbial composition comprised 24 studies focusing on single microorganisms and 3 studies on microbial consortia (Figure 6). The decrease rates in DI were 23.29–100% for single microorganisms and 15.45–75.95% for consortia. In DSI, the decrease ranges were 8.19–82.77% for single microorganisms and 43.29–60.32% for consortia. Combining the DI with the DSI, it is evident that single microorganisms exhibit better control for *Panax* plants. These findings support previous studies that have reported similar or even lower effectiveness in disease control when comparing applied microbial populations to single microorganisms [114,115,116]. Most of the available data on *Panax* plants are based on diverse, potted experiments. Therefore, in the future, a systematic comparison should be conducted to assess the biocontrol effectiveness of single microorganisms and microbial consortia within the same cultivation system. Additionally, the effectiveness of microbial control measures should be better tested in field conditions [117].

## 7. Conclusions

Soil-borne disease poses a significant threat to the quality and quantity of *Panax* plants. In this review, we initially explored the pathogenesis of soil-borne disease and the diversity of pathogens. Furthermore, we conducted a comprehensive review focusing on the diversity of biocontrol microbes, specifically bacteria, actinobacteria, and fungi. We examined their respective function mechanisms, emphasizing the synthesis of antagonistic substances, niche competition, and the induction of host resistance. In addition, we investigated the effects of microbial species, inoculation methods, inoculation volume, and microbial composition on the control efficiency of soil-borne diseases. We found that combining *Trichoderma* or *Burkholderia* with dipping or irrigation at higher inoculation volumes could lead to better results (Table 2). At present, research data related to *Panax* plants are limited, and more research data are needed in the future to verify the applicability of our conclusions to *Panax* plants.

## 8. Future Prospects

Although biological control has enormous potential benefits, its limitations cannot be ignored. The safety of the biological control microorganism is extremely important, due to the microorganism playing a crucial role in shaping disease outcomes in agriculture and having the potential for siderophore production by PGPR against a wide range of phytopathogens, making them an attractive and sustainable alternative to chemical fungicides and bactericides [118]. Moreover, when evaluating the effectiveness of biological control, the choice of quantitative indicators can significantly influence the results. In our opinion, DSI is a more recommendable evaluation criterion. Having sufficient data, it is crucial to perform a comprehensive analysis of the factors involved in biocontrol effectiveness in *Panax* plants. Examining the interaction among these factors and assessing the contribution of each can offer valuable insights for the application of biological control agents. Additionally, at present, there is a lack of specific research on nematodes in the prevention and control of disease in *Panax* plants. However, it has been reported that predatory nematodes can be vital in managing plant parasitic nematodes [119]. Therefore, future research should pay greater attention to this topic and further enrich investigations in this field.

## Figures and Tables

**Table 1:** Microbial biocontrol mechanisms.

Types	Strain	Inhibition Mechanisms	References
Bacteria	*Bacillus*	Synthesis of antagonistic substances; competition for ecological niches; induction of host resistance, reshaping the soil microbiome	[10,53,54,55,56]
	*Pseudomonas*	Competition for ecological niches, reshaping the soil microbiome	[54,56,57]
	*Burkholderia*	Synthesis of antagonistic substances	[50]
Fungi	*Trichoderma*	Synthesis of antagonistic substances; induction of host resistance	[58,59]
	*Chaetomium*	Competition for ecological niches	[51]
	*Penicillium*	Synthesis of antagonistic sub-stances	[52]
Actinomyces	*Streptomyces*	Synthesis of antagonistic substances; competition for ecological niches; induction of host resistance	[60,61,62]

**Table 2:** Suggested biocontrol strategies in this study.

Factor	Treatment	Biological Effect	Choice
Microbial species	*Bacillus*	DI/DSI	*Trichoderma/* *Burkholderia*
	*Pseudomonadaceae*		
	*Chaetomium globosum*		
	*Burkholderia*		
	*Brevundimonas*		
	*Lysobacter*		
	*Pseudomonas*		
	*Frankia*		
	*Trichoderma*		
	*Streptomyces*		
Inoculation method	Rootirrigation	DI/DSI	Root irrigation/root dipping
	Soil mixing		
	Root dipping		
	Foliar application		
	Foliar application plus root irrigation		
Inoculation volume	10^5^	DI/DSI	10^10^/10^8^
	10^6^		
	10^7^		
	10^8^		
	10^9^		
	10^10^		
Microbial composition	Single	DI/DSI	Single
	Consortia		
